# DNA defense systems become a fitness liability during plasmid conjugation

**DOI:** 10.1093/nar/gkag1043

**Published:** 2026-10-29

**Authors:** Jonathan N V Martinson, Leo C T Song, Benjamin E Rubin

**Affiliations:** Innovative Genomics Institute, University of California, Berkeley, CA 94720, United States; Innovative Genomics Institute, University of California, Berkeley, CA 94720, United States; Department of Comparative Biochemistry, University of California, Berkeley, CA 94720, United States; Innovative Genomics Institute, University of California, Berkeley, CA 94720, United States

## Abstract

Horizontal gene transfer via conjugative plasmids is a major driver of bacterial evolution. While antibiotic exposure selects for the resistance genes carried by some plasmids, much uncertainty remains about how plasmids persist and spread in microbial communities in the absence of such external selection. Here, we show that conjugative plasmid transfer selectively reduces the fitness of non-permissive recipients through the process of lethal zygosis: a phenomenon in which repeated Type IV Secretion System (T4SS) engagement damages recipients that fail to establish the plasmid. We found that this contact-dependent antagonism effectively clears a niche for plasmid donors by turning recipients’ DNA defenses into a liability. Blocking establishment of the plasmid RK2 with CRISPR–Cas or restriction-modification caused strong fitness defects in *Escherichia coli* recipients by preventing expression of plasmid-encoded exclusion genes and leaving cells exposed to repeated T4SS engagement. We leveraged this insight to resolve an obstacle in bacterial editing by developing transposon vectors that co-deliver exclusion genes, increasing the efficiency of gene editing by orders of magnitude. Our results reveal the role of “coercive assimilation” in horizontal gene transfer, exposing a tradeoff where the advantage of bacterial immunity to foreign DNA is counterbalanced by increased susceptibility to lethal zygosis.

## Graphical Abstract

**Graphical Abstract:**
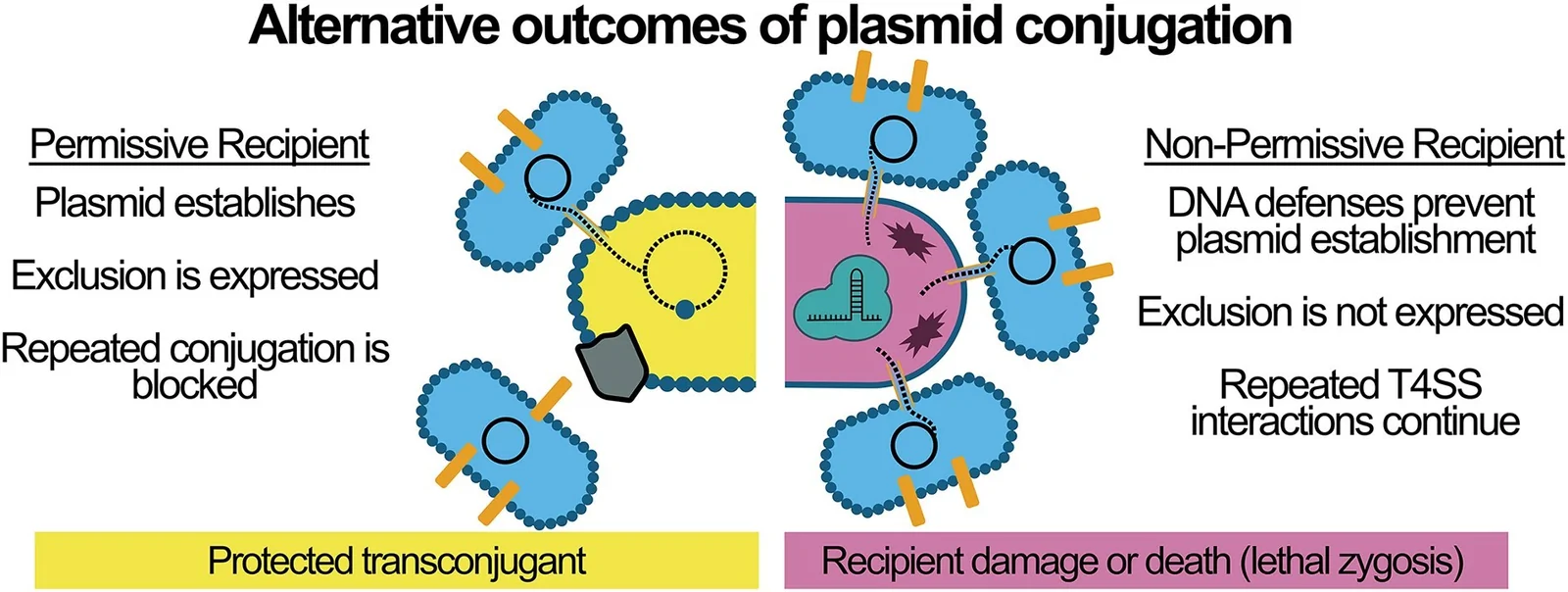

## Introduction

Conjugative plasmids drive many aspects of bacterial evolution by dispersing genetic information through microbial populations [1]. External selection, such as antibiotic use, can favor plasmid-encoded traits [2], but plasmid success also depends on factors that promote establishment, including rapid transmission and cargos that reduce the fitness costs of plasmid carriage [3]. Yet plasmid transfer does not always lead to stable establishment [4], and the consequences of these unsuccessful transfer events remain poorly understood.

To effectively spread, plasmids must prevent futile self-transmission [5, 6]. Cells carrying a conjugative plasmid therefore express plasmid-encoded genes that prevent redundant acquisition of the same plasmid [5, 7]. This process, known as plasmid exclusion, is primarily mediated by entry-exclusion genes and, to a lesser extent, surface-exclusion genes, which encode membrane proteins that inhibit the formation of productive mating pairs and DNA transmission (Fig. 1A) [5, 8, 9]. Entry-exclusion proteins are generally small, membrane-associated proteins that act specifically against their cognate conjugation systems [5, 10]. However, their precise molecular mechanisms are poorly understood. In the absence of exclusion proteins, continued engagement by the Type IV Secretion System (T4SS) can lead to multiple simultaneous mating events and cell death through membrane damage and/or an SOS response triggered by uncontrolled DNA influx (Fig. 1A) [6, 11, 12]. This conjugation-induced killing process, first described by Alfoldi, Jacob, and Wollman in 1957, is known as lethal zygosis [13]. While research has primarily focused on the mechanism of lethal zygosis using simplified experimental systems [6, 12–14], its broader implications for horizontal gene transfer have received little attention.

**Figure 1.**
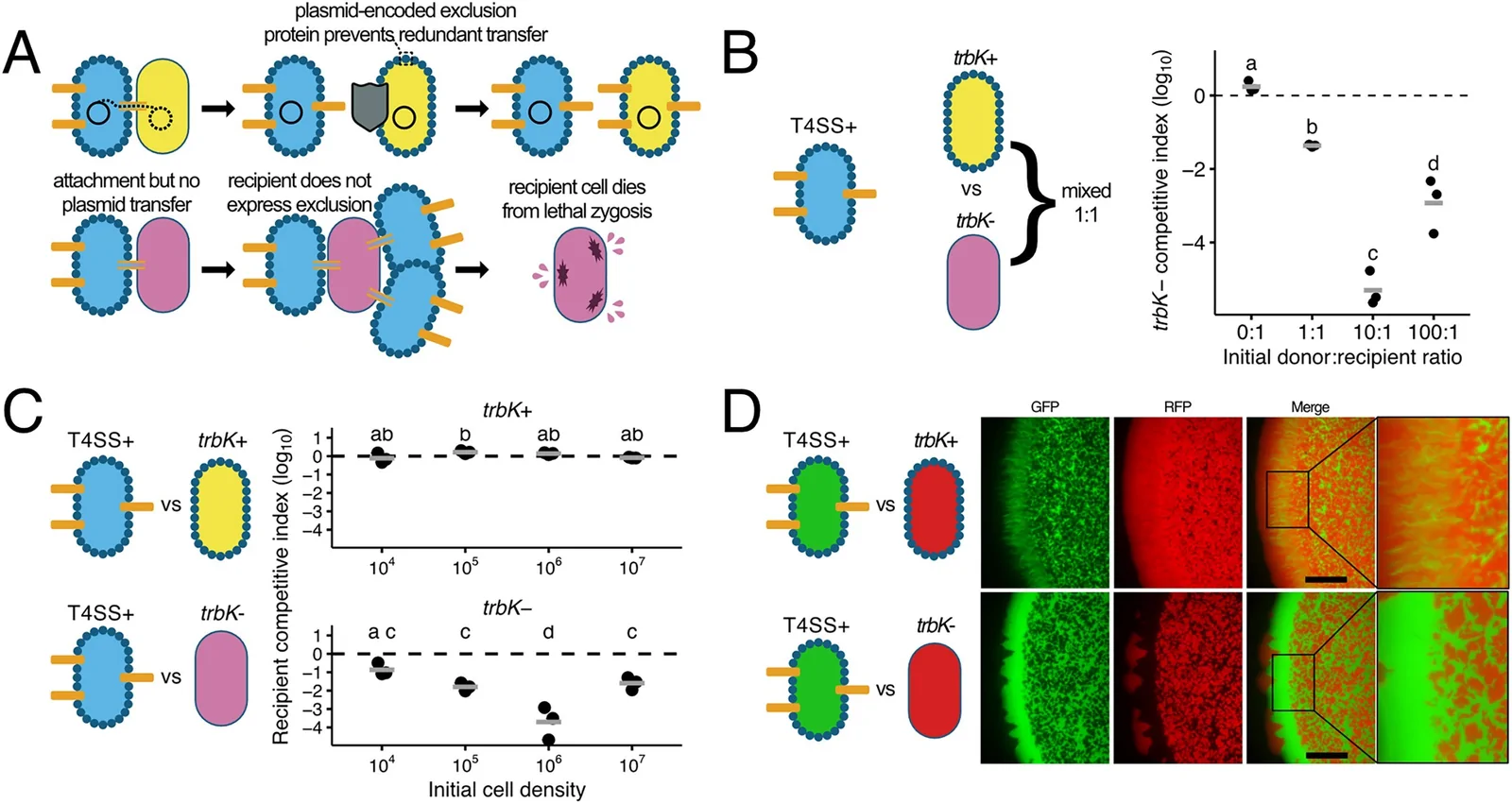
In the absence of plasmid transfer, lethal zygosis causes ratio- and density-dependent recipient fitness defects. (**A**) Diagram of plasmid conjugation and lethal zygosis. Top: In permissive recipients (yellow oval), plasmid establishment drives expression of the entry-exclusion factor TrbK (dark blue circles), creating a shielded state that blocks subsequent T4SS-mediated donor (light blue oval) contacts. Bottom: In situations where a cell expresses T4SS machinery, but does not transfer plasmid DNA, unshielded recipients are susceptible to repetitive, T4SS punctures (lethal zygosis). (**B**) Ratio-dependent fitness collapse. Left: Assay schematic. Plasmid-free donors (DATC) expressing T4SS were mixed with a 1:1 population of shielded (*trbK+*) and unshielded (*trbK–*) recipients. Right: Competitive index of the unshielded population (*trbK–*) relative to the shielded (*trbK*+) population mixed with varying amounts of plasmid-free donors. The dashed line indicates neutrality (CI = 0). Statistical significance was determined by one-way ANOVA followed by Tukey-adjusted pairwise comparisons. Different letters indicate statistically significant differences (*P* <.05). Circles represent the competitive index of replicates, horizontal bars represent the mean (*n* = 3). (**C**) Density-dependent fitness collapse. Left: Assay schematic. Plasmid-free donors were mixed 1:1 with either shielded (*trbK*+) or unshielded (*trbK–*) recipients across a range of initial cell densities (10^4 ^–10^7^ cells). Right: Unshielded recipients (*trbK–*, bottom) display reduced fitness (competitive index), whereas shielded recipients (*trbK*+, top) remain neutral across all densities. Statistical significance was determined by two-way ANOVA followed by Tukey-adjusted pairwise comparison. Different letters (a–d) indicate statistically significant differences (*P* <.05). (**D**) Spatial exclusion. Fluorescence microscopy of competition between GFP-labeled T4SS + donors (green) and RFP-labeled recipients (red). Shielded recipients (*trbK+*, top) intermix with donors, whereas unshielded recipients (*trbK–*, bottom) are spatially segregated. Scale bar, 750 µm.

In this study, we hypothesized that DNA defense systems targeting incoming conjugative plasmids can sensitize recipient cells to lethal zygosis by degrading plasmid DNA and thereby preventing the expression of exclusion systems. Consistent with this hypothesis, we found that recipients carrying either a restriction-modification (RM) system or CRISPR system targeting the broad host range plasmid RK2 (also known as RP4) had significantly reduced fitness relative to competing recipients without defense systems. These results suggest that conjugation confers a selective advantage on recipients that establish the plasmid and gain exclusion-mediated protection over those that fail to establish it and incur fitness costs from lethal zygosis. We term this fitness pressure to accept conjugative plasmids as “coercive assimilation.” We applied this insight to microbial editing, reasoning that standard mobilizable delivery vectors likely trigger lethal zygosis because they lack exclusion genes. We engineered a “shielded” delivery system carrying the RK2 entry-exclusion gene and demonstrated that this simple addition significantly improves editing efficiency for the randomly integrating Mariner transposon and the site-specific integrating *Vibrio cholerae* CRISPR-associated transposon (*Vch*CAST).

## Materials and methods

### Culture conditions

Liquid bacterial cultures were grown with shaking (200 rpm) in Terrific Broth (TB; Fisher Scientific, Cat. No. BP2468500) or lysogeny broth (LB; RPI, Cat. No. L24060-5000.0). For solid media, cells were cultured on lysogeny broth supplemented with 1.5% agar (BD, Cat. No. 214010). Experiments were performed at 37°C or 30°C, as needed. Where appropriate, antibiotics were added to liquid and solid media as follows: carbenicillin (100 µg/ml), chloramphenicol (34 µg/ml), gentamicin (10 µg/ml), kanamycin (25 µg/ml), and spectinomycin (100 µg/ml). Diaminopimelic acid (DAP) was added to media at 0.3 mM to support the growth of DAP-auxotrophic strains.

### Bacterial strains

The bacterial strains used in this study are listed in Supplementary Table S1. Cloning was primarily performed in *Escherichia coli* EC100D *pir*+ (Epicenter, Cat. No. 75927-934) and to a lesser extent BL21(DE3) (New England Biolabs, Cat. No. C2527H). Delivery of R6K suicide plasmids was performed using the DAP-auxotrophic strain DATC, which carries the RK2 conjugation machinery and R6K *pir* gene integrated into its chromosome [15]. For transposon delivery experiments, the following recipient strains were used: *E. coli* BW25113, *K. michiganensis* M5a1, and *Lysobacter* sp. OAE881.

### Plasmid construction

The plasmids used and generated in this study are listed in Supplementary Table S2. Routine cloning was performed using Gibson assembly (NEBuilder^®^ HiFi DNA Assembly Master Mix, New England Biolabs) from PCR-amplified fragments (Q5^®^ High-Fidelity DNA Polymerase, New England Biolabs). Plasmid assemblies were confirmed by Oxford Nanopore plasmid sequencing (Plasmidsaurus). For all *trbK* containing constructs, the gene was amplified from the RK2 plasmid and expression was driven by the medium-strength constitutive Anderson promoter (BBa_J23108) coupled with a ribosome binding site designed using the “Control Translation” function on denovodna.com, targeting a translation efficiency rate of 50 000 a.u. [16]. Plasmid pN3’s EcoRII RM system (NCBI reference sequence NC_015599.1) was synthesized (Twist Bioscience) based on coordinates described previously [17]. CRISPR–Cas9 guide RNAs were designed to target RK2’s oriV using the Benchling guide design tool [18]. The “shielded” transposon vectors were constructed by inserting RK2’s *trbK* inside the transposon repeats of previously developed Mariner transposon and *Vch*CAST vectors [19].

### Fluorescence microscopy

For spatial analysis (Fig. 1D), GFP-expressing T4SS+ cells (sJNVM0375) were mixed 1:1 with RFP-expressing strains with or without *trbK* (sJNVM0378 or sJNVM0390) at a low initial density (OD_600 _= 0.1). Mixtures (10 µl, ∼10^4^ cells) were spotted onto LB agar and incubated for 22 h at 37°C to allow growth. Colonies were imaged using a 4× objective lens on an EVOS M5000 imaging system (Thermo Fisher) using GFP and RFP fluorescence channels. For image processing, the individual fluorescence channels were merged and pseudocolored using Fiji software [20].

### General competition and mating assays

Unless otherwise noted, all competition and mating assays followed a standardized workflow.

*Preparation:* cells were grown in TB with appropriate selection. For experiments in Fig. 1, stationary phase cells from overnight cultures were used directly. For experiments in Figs. 2 and 3, overnight cultures were back-diluted and grown to mid-log phase prior to harvest. *Washing and Normalization:* Cells were washed three times in phosphate-buffered saline (PBS) at 3500× g. Recipients were normalized to OD_600 _= 1.0 (see exceptions below). In cases where two recipient strains were competed, strains were mixed at a 1:1 ratio. We resuspended cells by pipetting instead of vortexing to avoid damaging the conjugative apparatus. *Mating:* To establish the various experimental conditions, the normalized donor and recipient cultures were combined to achieve the various specific donor-to-recipient ratios required for each assay. For each unique strain combination and ratio, 10 µl aliquots of the mixture were spotted onto LB agar (supplemented with DAP for auxotrophs) to form distinct, physically separated conjugation (or competition) spots. Multiple conjugation spots were prepared from the same independently grown cultures and averaged to obtain a single biological replicate. The spots were allowed to dry near a flame prior to incubation (∼5 min ). *Harvest:* After incubation at 37°C for 5 h, spots were scraped into 100 µl PBS using sterile loops, serially diluted, and plated on appropriate media.

Specific experimental variations are detailed below:

Ratio-dependent lethal zygosis and donor crowding control (Fig. 1B and Supplementary Fig. S1): *trbK–* and *trbK*+ EC100D recipients were mixed 1:1 and normalized to OD_600_ = 1. DATC donors carrying the chromosomally integrated RK2 T4SS were adjusted to varying densities (OD_600_ = 1, 10, and 100) and combined with the recipient mixture to establish the indicated donor-to-recipient ratios (Fig. 1B). As a donor crowding control, the same experiment was performed using a matched EC100D pir + donor-background strain lacking a T4SS (Supplementary Fig. S1).

Density-Dependent Survival (Fig. 1C): DATC donors and recipients (*trbK+* or *trbK–*) were normalized to OD_600_ 100, mixed 1:1, and serially diluted in PBS prior to spotting to achieve initial spot densities of 10^7^ –10^4^ cells (total cells per spot).

Restriction-Modification Experiments (Fig. 2B and D,  Figs. S2 and  S3): BL21(DE3) was used as the base strain for both donors and recipients, as it is Dcm-negative (EcoRII modifies the same motif), allowing us to control RK2 methylation by providing the EcoRII methylase *in trans* on a separate plasmid in the donor. Donors carrying RK2 with the methylase plasmid produced methylated RK2 (protected from EcoRII digestion); donors lacking the methylase plasmid produced unmethylated RK2. Recipients either carried the full EcoRII restriction-modification system (RM+) or an empty vector control (RM–) with differential antibiotic resistance markers; see Supplementary Table S2. For Fig. 2B, RM+ and RM– recipients were mixed 1:1 and competed against methylated or unmethylated RK2 donors at 0:1, 1:1, 5:1, and 10:1 donor-to-recipient ratios. For Fig. 2D, the same assay was performed with RM+ recipients additionally expressing *trbK*. For Supplementary Fig. S2, RM+ and RM– recipients carrying the *parABCDE* operon were mixed 1:1 and competed against methylated or unmethylated RK2 donors at 0:1 and 5:1 donor-to-recipient ratios. For Supplementary Fig. S3, unmethylated or methylated RK2 donors were the focal strain and competed with RM+ recipients at 1:10 and 1:1 donor-to-recipient ratios.

Conjugation Time Course (Supplementary Fig. S4): RM+ or RM– recipients were mixed with either donors carrying unmethylated RK2 or matched donor-background cells [BL21(DE3)] lacking RK2 at an initial donor-to-recipient ratio of 10:1. Conjugations were performed on solid LB agar as described above. Conjugation spots were harvested hourly over a 5 h period and plated to enumerate viable recipients and transconjugants. This experiment was performed as a single biological replicate, with three conjugation spots destructively sampled at each time point.

CRISPR–Cas9 Induction Experiments (Fig. 2C): Recipient cultures were split into induced (100 ng/ml anhydrotetracycline [aTc]) and uninduced conditions and incubated for 1 h at 37°C prior to washing. Mating spots were plated on LB agar supplemented with aTc where appropriate. In Fig. 2C, recipients carrying CRISPR–Cas9 plasmids either targeting or not targeting RK2 were competed in the presence or absence of an RK2 donor strain at 0:1, 1:1, 5:1, and 10:1 donor-to-recipient ratios.

Shielded Transposon Delivery (Fig. 3 and Supplementary Fig. S5): Donors (OD_600_ 1 or 10) and recipients (OD_600_ 1.0) were mixed at 1:1 or 10:1 ratios. Conjugation spots were incubated at 30°C for 5 (*E. coli* BW25113 and *K. michiganensis* M5a1) or 18 h (*Lysobacter* sp. OAE881). Cells were plated on selective media (LB supplemented with chloramphenicol) to enumerate transposition and plain LB for total survival. For *Vch*CAST experiments, the *Vch*CAST operon was expressed from either a constitutive Plac or repressible Ptet promoter. For the Ptet system, TetR was expressed in donor cells from a separate non-mobilizable plasmid, thereby repressing *Vch*CAST expression prior to conjugation. Because the TetR-expressing plasmid was not mobilized with the *Vch*CAST vector, Ptet-driven *Vch*CAST expression was derepressed following transfer into recipient cells. *Vch*CAST vectors targeted the previously validated neutral intergenic site described by Rubin *et al*. [21].

### Calculation of competitive index

To quantify the relative fitness of a focal strain versus a reference strain, the competitive index was calculated as the ratio of focal to reference strain at the end of the experiment divided by the corresponding ratio at the beginning of the experiment. Competitive index values were then log_10_ transformed. Accordingly, a log_10_ competitive index of zero indicates no change in relative abundance, whereas positive or negative values indicate increased or decreased relative fitness of the focal strain, respectively.

### Data visualization and statistical analysis

Data visualization and calculations were performed using R v4.4.0 with the tidyverse [22]. Statistical analyses and post-hoc comparisons were performed using the emmeans package [23]. Final figures were assembled and refined using Affinity v3.0.2.3912 (Canva). Competitive index values and cell population counts were log_10_ transformed and a pseudocount was applied to handle zero values. When technical replicates were collected, these were averaged within each independent biological replicate prior to statistical analysis. Statistical comparisons were performed using one-way, two-way, or three-way ANOVA as appropriate for the experimental design, including interaction terms between experimental factors. Significant effects and interactions were further evaluated using estimated marginal means with Tukey- or Holm-adjusted contrasts, as specified for each analysis. An adjusted *P*-value of <.05 was considered statistically significant.

## Results

### Lethal zygosis is ratio- and density-dependent

To validate the role of conjugation machinery in driving lethal zygosis, we established a competitive fitness assay comparing recipients with and without the RK2 plasmid entry-exclusion factor, TrbK [8]. TrbK is a 69-residue inner-membrane lipoprotein that acts in recipient cells to inhibit redundant transfer of RK2 [8, 24]. To isolate the physiological effects of the mating pair formation complex from secondary effects of plasmid transfer on the recipient, we used the donor strain DATC, which carries RK2’s conjugation machinery genes integrated into its chromosome [15]. Although we refer to our DATC strain as a donor, it does not transmit DNA unless transformed with a plasmid carrying an origin of transfer (oriT). This system allowed us to decouple the mechanical process of conjugation from DNA transfer, isolating the specific impact of entry exclusion on recipient fitness.

We first examined the ratio (i.e. frequency) dependence of lethal zygosis by mixing recipients with or without RK2’s entry-exclusion gene *trbK* at a 1:1 ratio and competing these recipients in the presence of increasing amounts of DATC donor cells (Fig. 1B). Similar to other studies [12, 14], we observed a significant effect of donor-to-recipient ratio on the fitness of recipients lacking *trbK* (one-way ANOVA, F(3, 8) = 84.58, *P* < .001). While the competitive index of the *trbK–* population remained close to neutral in the absence of donors, it declined precipitously with increasing donor-to-recipient ratio, reaching a minimum at a 10:1 ratio (Fig. 1B). To determine whether the reduced fitness of *trbK–* recipients could result from donor-cell crowding alone, we repeated the competition using a matched donor-background strain lacking the T4SS. Increasing the initial donor-to-recipient ratio had no detectable effect on the relative fitness of *trbK–* recipients (one-way ANOVA, F(3, 8) = 0.076, *P* = 0.971; Supplementary Fig. S1), indicating that donor-cell crowding alone is insufficient to produce the fitness defect and supporting a specific role for the T4SS in reducing the fitness of recipients lacking entry exclusion.

Because conjugation is a contact-dependent process [25], we hypothesized that lethal zygosis would also be governed by cell density, a relationship that, to our knowledge, has not been previously demonstrated. To test this, we performed competition assays between DATC donors and either *trbK*+ or *trbK–* recipients across a range of initial cell densities (10^4^ –10^7^ cells per conjugation spot), while holding the initial donor-to-recipient ratio constant (1:1) (Fig. 1C). We observed a significant interaction between recipient genotype and cell density (two-way ANOVA, F(3, 16) = 17.99, *P* < .0001), confirming that DATC donor crowding selectively impacts populations without *trbK*. Recipient fitness partially recovered at the highest density, similar to the recovery at the highest donor-to-recipient ratio in Fig. 1B. Although conjugation rates saturate at high cell densities [25], the cause of this recovery remains unclear. Limited space or nutrients may have constrained population expansion, reducing the opportunity for fitness differences to accumulate. Nevertheless, while *trbK*+ recipients remained protected across all densities (Fig. 1C, top panel), *trbK–* recipients exhibited density-dependent fitness defects, with the largest reduction at 10⁶ cells per conjugation spot (Fig. 1C, bottom panel).

Finally, we visualized colony-scale spatial dynamics of this antagonism using fluorescence microscopy. We mixed GFP-labeled DATC donors with RFP-labeled recipients on solid agar. When donors were mixed with protected recipients (*trbK+*), the populations intermixed freely. However, mixtures with unprotected recipients (*trbK–*) revealed distinct spatial segregation, characterized by a sharp boundary between the donor and recipient populations (Fig. 1D), similar to boundaries produced by other contact-dependent killing weapons (e.g. Type VI secretion systems, CDI) [26, 27]. We hypothesize that this boundary results from reduced fitness of *trbK–* recipients at sites of frequent donor-recipient contact, restricting their intermixing with the donor population, although the cellular dynamics underlying this spatial pattern remain unresolved.

### Bacterial immunity creates a fatal liability during conjugation

DNA defense systems, such as RM and CRISPR–Cas, are often characterized as fitness-enhancing elements that protect cells from hostile mobile genetic elements (MGEs) like bacteriophage [28]. However, avoiding lethal zygosis from a conjugative donor requires receipt of the plasmid and expression of the encoded exclusion genes. We reasoned that if bacterial immune defenses degrade the incoming DNA before these genes can be expressed, active defense would create a fitness vulnerability: by blocking plasmid establishment, the host fails to acquire the protective “shield” and is consequently eliminated by lethal zygosis (Fig. 2A).

**Figure 2.**
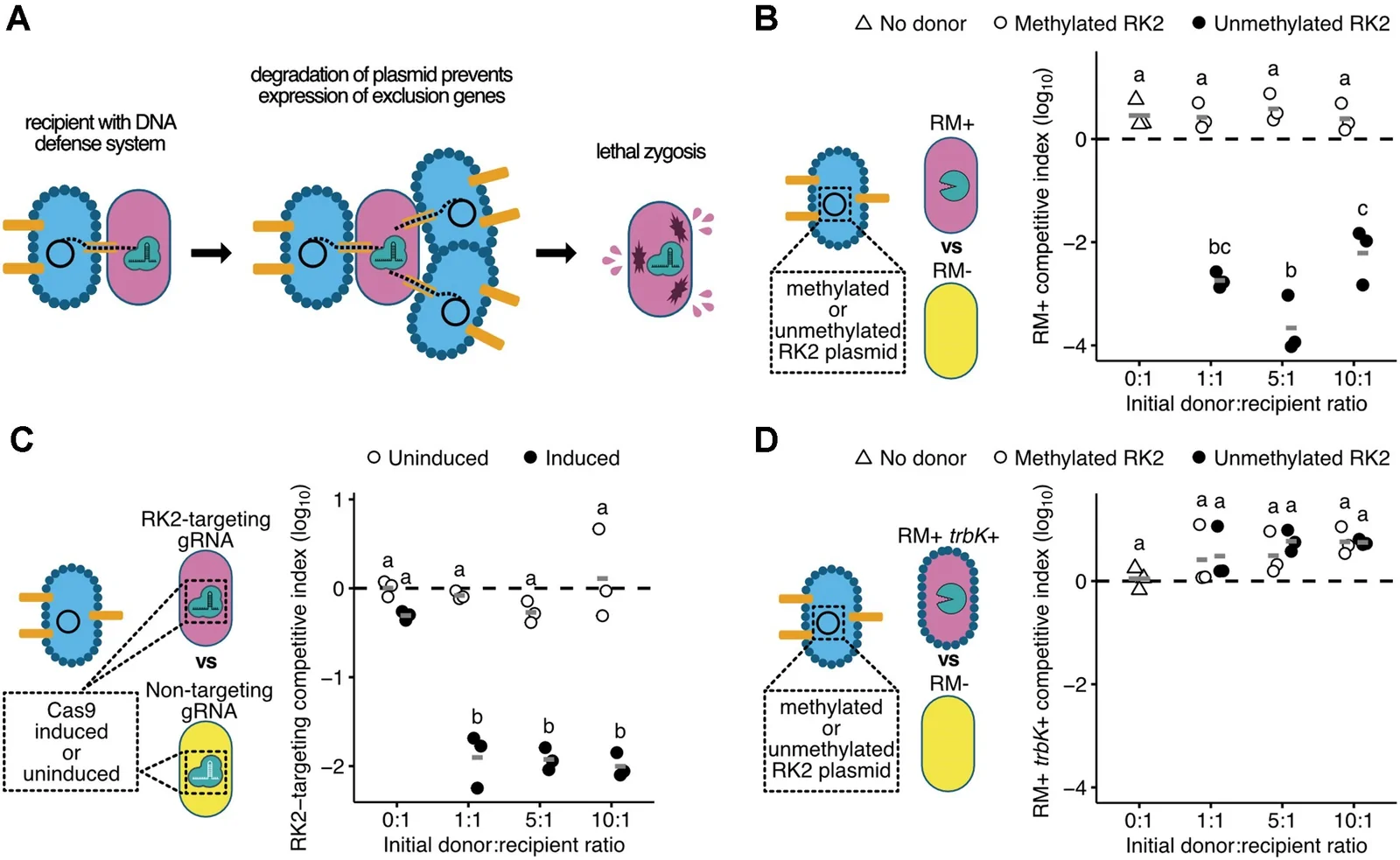
Bacterial immune systems prevent acquisition of exclusion genes, leading to non-permissive recipient fitness reduction via lethal zygosis. (**A**) Model of coercive assimilation. In non-permissive recipients (pink oval), intracellular defense systems (e.g. CRISPR–Cas9, teal) degrade the incoming plasmid, preventing TrbK expression. The unshielded recipient remains susceptible to repetitive T4SS punctures and uncontrolled DNA delivery (lethal zygosis). (**B**) RM defense. Left: Schematic of the RM competition assay. RM+ recipients (non-permissive, pink) and RM*–* recipients (permissive, yellow) were competed in the presence of varying amounts of donor cells carrying RK2 plasmids (blue) that are either methylated (protected from RM digestion) or unmethylated (susceptible to RM digestion). Right: competitive index of RM+ recipients relative to RM*–* recipients. Triangles indicate the no-donor control, open circles indicate methylated RK2, and filled circles indicate unmethylated RK2. (**C**) CRISPR–Cas defense. Left: Schematic of the CRISPR competition assay. Recipients carrying Cas9 and an RK2-targeting gRNA (non-permissive, pink) or a non-targeting gRNA (permissive, yellow) were competed in the presence of varying amounts of RK2 donor cells, with Cas9 either induced or uninduced. Open circles indicate uninduced Cas9 and filled circles indicate induced Cas9. (**D**) TrbK rescue of RM-associated fitness loss. Left: schematic of the rescue competition assay. RM+ recipients pre-expressing the RK2 entry-exclusion gene *trbK* (pink) were competed against RM*–* recipients (yellow) in the presence of donors carrying either methylated or unmethylated RK2. Right: Competitive index of RM+ TrbK+ relative to RM*–* recipients. Symbols are as in panel (B). For all panels, points represent biological replicates and grey bars indicate means (*n* = 3). Different letters indicate significantly different groups (Tukey-adjusted pairwise comparisons, *P* <.05); groups sharing a letter are not significantly different.

To test this hypothesis, we first investigated the effects of the RM system, EcoRII, that prevents establishment of RK2 during conjugation [4]. We competed RM+ recipients (non-permissive to plasmid establishment) against recipients lacking the restriction enzyme (permissive). Crucially, we modulated the “visibility” of the incoming plasmid to the RM system by using donors carrying either methylated (invisible) or unmethylated (visible) RK2 plasmids. We observed a significant interaction between donor methylation status and the donor-to-recipient ratio across donor-present conditions (Fig. 2B: two-way ANOVA, F [2, 12] = 7.62, *P* = 0.0073), indicating that restriction activity alters the outcome of the competition. When donors carried methylated plasmids, which evade EcoRII digestion, the RM+ recipients were not disadvantaged relative to RM– recipients, with donor exposure having no significant effect on their relative fitness compared with the no-donor control (estimated marginal means contrast, Holm-adjusted *P* = 1.0 at all donor-to-recipient ratios, Fig. 2B). In contrast, when donors carried unmethylated plasmids, the RM+ recipients were strongly selected against, with their competitive index reduced by ∼3 to 4 orders of magnitude relative to the no-donor condition across all tested donor-to-recipient ratios (estimated marginal means contrasts, Holm-adjusted *P* <.0001 at all donor-to-recipient ratios). Thus, restriction-mediated defense against RK2 establishment becomes a competitive disadvantage.

To determine if this effect extended to other defense systems, we performed a competitive fitness assay with recipients carrying an inducible CRISPR–Cas9 (Fig. 2C). We competed recipients expressing either a non-targeting guide RNA (gRNA; permissive) or a gRNA targeting the RK2 origin of replication (non-permissive) in the presence of varying amounts of RK2 donor cells. We observed a significant interaction between Cas9 induction and donor-to-recipient ratio (two-way ANOVA, F (3, 16) = 18.96, *P* < .0001). Consistent with the RM results, the fitness of the non-permissive recipient population collapsed in the presence of donors when Cas9 was induced with a ∼40 to 50-fold reduction in fitness relative to the induced no-donor control across all tested donor-to-recipient ratios (estimated marginal means contrasts, Holm-adjusted *P* <.0001 at all ratios).

To directly test whether exclusion protects immune non-permissive recipients from this fitness defect, we pre-expressed the RK2 entry-exclusion gene *trbK* in RM+ recipients and repeated the competition shown in Fig. 2B. In contrast to RM+ recipients lacking *trbK*, RM+ TrbK+ recipients maintained high competitive fitness when challenged with either methylated or unmethylated RK2 (Fig. 2D). Competitive fitness was unaffected by donor plasmid methylation or donor-to-recipient ratio across donor-present conditions (two-way ANOVA, *P* ≥ .05), and donor exposure did not significantly alter competitive fitness relative to the no-donor control at any tested ratio (estimated marginal means contrasts, Holm-adjusted *P* ≥ .173). Thus, pre-expression of TrbK rescued the restriction-associated fitness defect observed in Fig. 2B, supporting a model in which failure to acquire plasmid-encoded exclusion systems sensitizes immune recipients to lethal zygosis.

Notably, for these experiments (Fig. 2), the magnitude of fitness reduction in the RK2-targeting recipients remained relatively consistent regardless of donor-to-recipient ratio. These results differed from the plasmid-free donor experiments, where fitness of recipients varied with donor-to-recipient ratio (Fig. 1B) or initial cell density (Fig. 1C). To explore alternative mechanisms that could contribute to the restriction-associated fitness defect, we tested whether degradation of incoming RK2 activated its parDE toxin-antitoxin (TA) system. We pre-expressed the RK2 parABCDE operon in recipient cells, similar to the approach used by Sünderhauf *et al*. to prevent TA-mediated killing following RK2 loss [29]. Despite pre-expression of antitoxin from the *parABCDE* operon, RM+ recipients remained strongly disadvantaged when challenged with unmethylated RK2, with a ∼3.6-log_10_ reduction in competitive fitness relative to recipients challenged with methylated RK2 (estimated marginal means contrast, Holm-adjusted *P* < .0001; Supplementary Fig. S2). These results argue against activation of the *parDE* TA system as the primary cause of the restriction-associated fitness defect.

Contact-dependent weapons, such as T6SS, often provide bacteria the capacity to invade communities when initially rare [27]. To test if lethal zygosis provides a similar competitive advantage, we competed RK2 donor strains carrying either methylated or unmethylated plasmids against an RM+ recipient at varying initial ratios (Supplementary Fig. S3A). When donors carried the methylated (protected from digestion) plasmid, donor fitness remained similar (near neutral) between initial ratios of 1:10 and 1:1 (Supplementary Fig. S3B). In contrast, the fitness of donors carrying unmethylated RK2 depended strongly on their initial frequency. When initially rare (1:10), unmethylated donors were selected against, with a competitive index of approximately −0.8 log_10_ (Supplementary Fig. S3B). This suggests that, when the donor is rare, the burden of plasmid carriage may outweigh the potential fitness advantages of lethal zygosis. However, this dynamic inverted when the initial ratio was 1:1, as unmethylated plasmid donors’ competitive index increased to ∼2.6 log_10_ (Supplementary Fig. S3B). Consistent with these contrasting effects, donor plasmid methylation and initial donor-to-recipient ratio showed a significant interaction [two-way ANOVA, F(1, 12) = 184.17, *P* < .0001]. These data suggest that the fitness effects associated with defense system-induced lethal zygosis exhibit strong frequency dependence.

Because the 5 h conjugation assays allow substantial time for population growth, plasmid acquisition, and secondary transfer, we next examined the temporal dynamics underlying these endpoint measurements. We performed a time-course experiment with RM+ and RM– recipients exposed to donors carrying unmethylated RK2 or matched donor-background cells lacking the plasmid (Supplementary Fig. S4). In the absence of RK2, both recipient populations increased throughout the 5 h experiment. RM– recipients continued to increase following exposure to RK2, while transconjugants progressively accumulated and approached the total recoverable recipient population at later time points. In contrast, RM+ recipients exposed to RK2 initially increased during the first 2 h but subsequently declined, falling below their initial abundance by 5 h; no transconjugants were detected throughout the experiment. Thus, the fitness differences measured after 5 h in Fig. 2 emerge over several hours of conjugation, during which both recipient abundance and the fraction of transconjugants carrying RK2 can change substantially.

### Exclusion genes improve genetic editing efficiency

Delivery of non-replicative plasmids (i.e. suicide plasmids) is essential for forward-genetics approaches such as transposon mutagenesis, allelic exchange mutagenesis, and potentially *in vivo* microbiome editing, where the use of replicative vectors encoding editors raises concerns about uncontained dissemination of MGEs [21, 30, 31]. However, the delivery of non-replicative vectors via conjugation often results in low efficiencies, especially in non-model organisms [21, 32]. We hypothesized that this poor performance is partially attributable to lethal zygosis. Because engineered mobilizable plasmids lack exclusion genes, they render transconjugants susceptible to uncontrolled conjugation and subsequent death. To test this, we integrated RK2’s entry-exclusion gene, *trbK*, into the payload of the randomly integrating Mariner transposon system [30, 33]. We reasoned that by providing recipient strains a “shield” against lethal zygosis in the form of an exclusion gene, we would increase editing yield by preventing conjugation-induced death (Fig. 3A).

**Figure 3.**
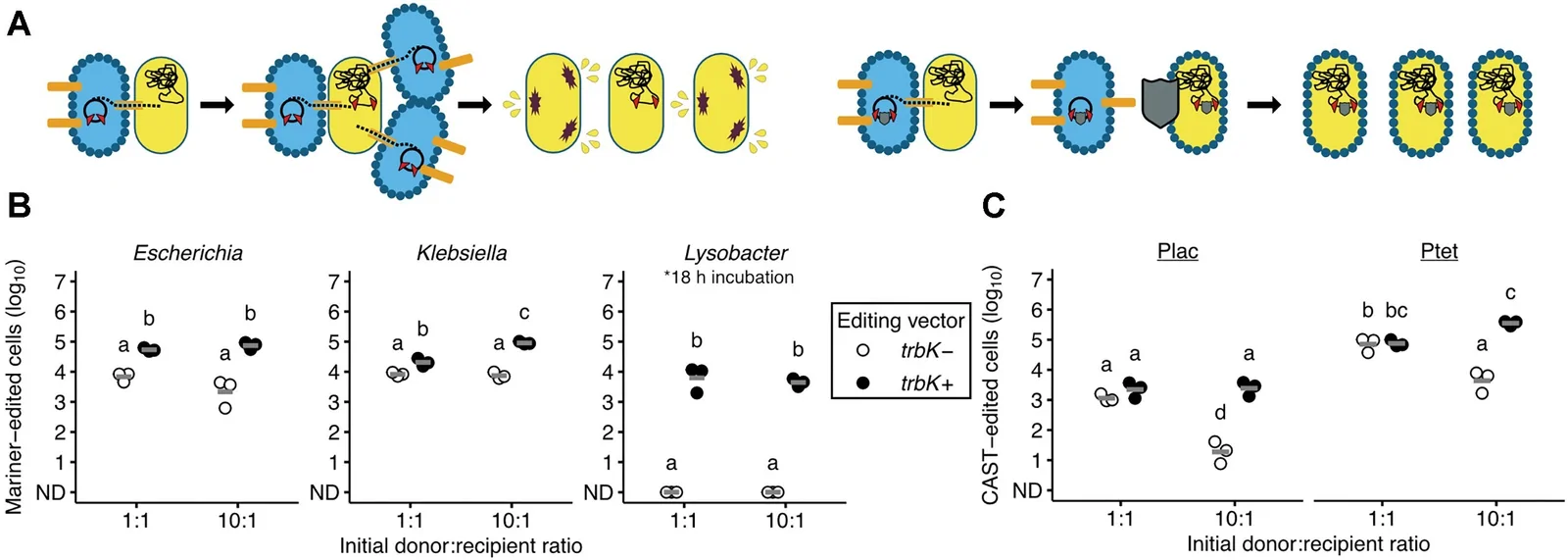
Inclusion of exclusion genes improves editing yield across transposon systems. (**A**) Schematics illustrating the mechanism of lethal zygosis during standard transposon delivery (left) versus the proposed model of how co-delivery of an exclusion gene improves editing yield by shielding recipient cells from repeated T4SS engagement (right). (**B**) Mariner transposon editing yield following conjugation with *Escherichia, Klebsiella*, and *Lysobacter* recipients. All conjugations were performed for 5 h, with the exception of Lysobacter, which required 18 h to yield detectable edited cells (no edits were observed at 5 h). (**C**) *Vch*CAST transposon editing yield in *Escherichia* recipients under the control of two promoters (Plac, Ptet). Conjugations were performed for 5 h. “ND” indicates that no colonies were detected; values of zero were assigned a pseudocount of one prior to log₁₀ transformation for visualization. Filled circles: editing vector with *trbK*; open circles: vector without *trbK*. For panel (B), data were analyzed separately for each recipient species by two-way ANOVA testing the effects of *trbK*, donor-to-recipient ratio, and their interaction. For panel (C), data were analyzed by three-way ANOVA testing the effects of *trbK, Vch*CAST promoter, donor-to-recipient ratio, and their interactions. Different letters indicate significant Tukey-adjusted pairwise differences among conditions (*P* <.05); for panel (C), comparisons include all Plac and Ptet conditions shown. Horizontal bars indicate the mean of biological replicates (*n* = 3).

We found that delivering standard vectors (lacking *trbK*) at high donor-to-recipient ratios (10:1) caused a significant reduction in the number of viable recipients in both *Escherichia* and *Lysobacter* (Supplementary Fig. S5A). Co-delivering the *trbK* gene within the Mariner transposon payload effectively prevented this donor-density-dependent loss of recipients in these strains, allowing the “shielded” vectors to maintain high recipient viability at both 1:1 and 10:1 ratios (*P* < .05, two-way ANOVA followed by Tukey-adjusted pairwise comparisons). Consequently, this protective effect translated into improved editing yields, with *trbK*-containing vectors consistently generating significantly more edited cells *across Escherichia, Klebsiella*, and *Lysobacter* (Fig. 3B, *P* < .05, two-way ANOVA followed by Tukey-adjusted pairwise comparisons). We found this advantage to be most notable in *Lysobacter*: while a 5 h conjugation yielded no edited cells, extending the assay to 18 h resulted in edited cells exclusively with the *trbK*-shielded vector. Notably, the “shielding” effect in *E. coli* at the 10:1 ratio resulted in an ∼1000-fold increase in recipient survival while increasing the editing yield by ∼34-fold. Given that the increase in survival was substantially greater than the increase in editing, these results suggest that transient expression *of trbK* can function as a population-wide shield against lethal zygosis, preserving recipients even without stable integration of the transposon.

We next assessed whether the inclusion of *trbK* improves editing yield for the targeted integration tool, *Vch*CAST. To test this, we delivered *Vch*CAST transposon vectors into *E. coli* BW25113 recipients targeting a previously validated intergenic site with neutral fitness effects [21]. Using *Vch*CAST driven by a constitutively expressed promoter (Plac), we found that *trbK* protected recipient cells from lethal zygosis at 10:1 donor-to-recipient ratios (three-way ANOVA, *trbK* x donor-to-recipient ratio: F (1, 16) = 213.54, *P* < .0001; Supplementary Fig. S5C). At the 10:1 ratio, Plac-driven *Vch*CAST without *trbK* resulted in reduced editing yield compared to the 1:1 ratio, whereas the Plac-driven *Vch*CAST carrying *trbK* showed no difference in editing yield between ratios (Fig. 3C). We suspected that providing a stronger promoter for the *Vch*CAST machinery would increase editing yield. To prevent the toxicity associated with high *Vch*CAST expression in the donor cell, we developed a split-repression strategy similar to those used in fluorescence reporter systems to track conjugation [34–36]. We placed the *Vch*CAST system under the control of a stronger Ptet promoter and repressed it in the donor using a *tetR* repressor gene located on a separate, non-mobilizable plasmid (Supplementary Fig. S5B). Because the *tetR* repressor is not co-delivered during conjugation, *Vch*CAST is strongly expressed from Ptet only after transfer into the recipient cell. The split-Ptet promoter system increased editing yield relative to Plac across both donor-to-recipient ratios (Fig. 3C; promoter effect: F(1, 16) = 380.82, *P* < .0001), without significantly affecting recipient survival (Supplementary Fig. S5C; promoter effect: F(1, 16) = 1.03, *P* = 0.324). At the 10:1 ratio, inclusion of *trbK* further increased Ptet-driven editing yield ∼81-fold, producing the highest editing yield observed (Fig. 3C). Together, recipient-specific *Vch*CAST expression and protection from lethal zygosis increased editing yield by approximately four orders of magnitude relative to the standard unshielded Plac system at high donor density.

## Discussion

In this study, we demonstrate that plasmid conjugation imposes severe fitness costs on recipients that resist plasmid acquisition. Specifically, we show that when cells mount an active defense against plasmids, they are sensitized to lethal zygosis because they degrade the DNA encoding exclusion proteins that protect cells from uncontrolled, repeated conjugation events (Fig. 2). In this way, conjugative plasmids provide a strong selective pressure for their own engraftment.

This mechanism creates a tradeoff whereby bacterial immune systems that are often critical for surviving lytic phage pressure [28] become a liability during conjugation. These countervailing forces could feasibly establish a cyclic rock-paper-scissors dynamic [37]: Phage pressure selects for immunity; immunity sensitizes bacteria to lethal zygosis, increasing population permissiveness; and this permissiveness resets the cycle by exposing the population to phage predation once again (Fig. 4). Although this is a simplified model, it provides a mechanistic rationale for the observation that CRISPR systems target plasmids far less frequently than bacteriophages, despite the metabolic burden plasmids impose [38]. If targeting a plasmid induces lethal zygosis, anti-plasmid spacers would be actively selected against. Consistent with this, restriction–modification systems can restrict phage with orders of magnitude more efficacy than conjugative plasmids [4, 39]. While these observations do not imply causation, they align with a model in which coercive assimilation creates a selective pressure against plasmid targeting while preserving phage defense.

**Figure 4.**
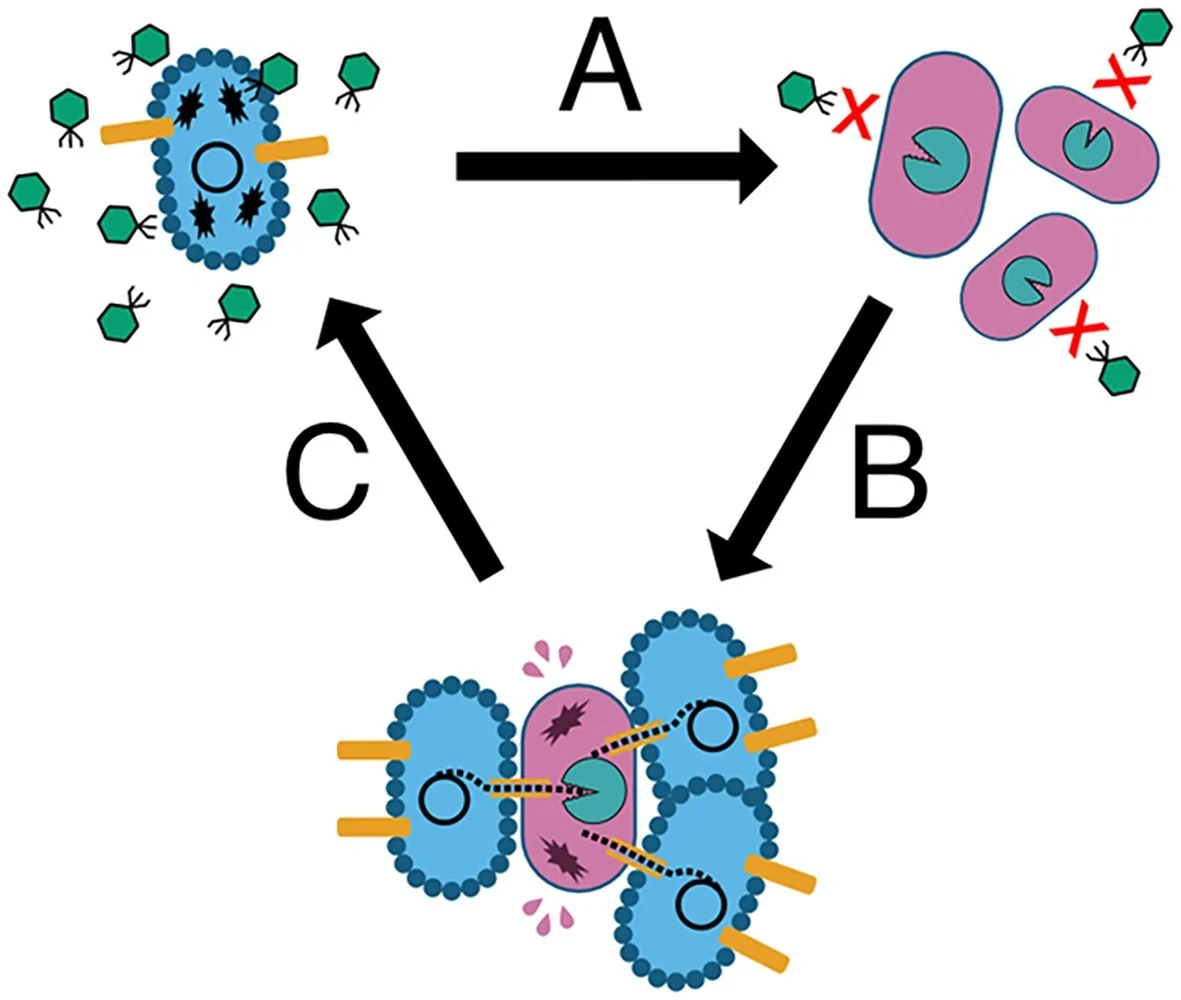
A “Rock-Paper-Scissors” evolutionary dynamic driven by the incompatibility of defense and conjugation. (**A**) Phage pressure selects for immunity by eliminating susceptible permissive hosts. (**B**) Lethal zygosis selects against immunity, as cells lacking active defense survive plasmid uptake and acquire exclusion genes. (**C**) This permissive state resets the cycle by exposing the population to phage predation once again.

The ecological consequences of lethal zygosis likely extend beyond RK2 and *E. coli*. For example, lethal zygosis has been observed in *Bacillus subtilis* following the loss of the ICEBs1 exclusion gene, *yddJ* [40, 41]. Additionally, recent work has shown that RK2 conjugation machinery is capable of causing interspecies lethal zygosis against diverse members of Enterobacterales and Pseudomonadales [12]. These effects may therefore extend across diverse MGEs and bacterial taxa. However, this mechanism requires conjugative machinery and is therefore limited to conjugative plasmids and other conjugative elements. Conjugative plasmids comprise ∼25% of known plasmids [42, 43], and exclusion systems are widespread among conjugative elements, although their high sequence divergence often makes them difficult to identify [5].

The magnitude of coercive assimilation is likely to depend not only on the host range of the conjugation machinery but also on interactions between recipient defense systems and plasmid counter-defense mechanisms. RM systems, for example, vary by several orders of magnitude in their ability to restrict conjugative plasmids, with restriction generally increasing with the number of cognate recognition sites in the incoming plasmid [4]. Because our results indicate that degradation of incoming DNA can prevent establishment of exclusion and thereby sensitize recipients to lethal zygosis, such variation in defense activity may strongly influence susceptibility. Conversely, conjugative plasmids frequently encode counter-defense functions, including anti-restriction and anti-CRISPR proteins, which are enriched in the leading region and can act early during plasmid establishment [44, 45]. These functions could reduce recipient vulnerability by promoting plasmid establishment and subsequent expression of exclusion genes. The occurrence and magnitude of these effects may therefore emerge from the combined specificity of donor–recipient interactions, the activity of recipient defenses, and the ability of the incoming element to evade those defenses.

The host range of conjugative systems may determine the ecological breadth of coercive assimilation. Broad-host-range plasmids such as RK2 may affect a large fraction of a microbial community, whereas host-restricted systems, such as IncF plasmids, may concentrate their effects within particular taxa [35, 36, 46, 47]. Importantly, the taxonomic range over which a conjugative system physically engages cells may exceed the range over which its plasmid can successfully establish [47, 48]. Thus, differences among conjugative systems may determine not only how frequently these fitness effects occur, but also which members of a microbial community experience them.

By selectively disadvantaging non-permissive recipients, lethal zygosis could favor plasmid persistence by increasing the relative fitness of plasmid-bearing cells. Our experiments establish this mechanistic and ecological effect but do not demonstrate that lethal zygosis evolved as an adaptation for interbacterial competition [49]. The potential benefit to plasmid-bearing populations also depends on population context. Plasmid donors were disadvantaged when initially rare under the conditions tested (Supplementary Fig. S3), whereas lethal zygosis may favor established plasmid-bearing populations by suppressing invading non-permissive competitors, analogous to colonization resistance. This effect may be particularly relevant in high-density ecosystems such as biofilms, where frequent cell-to-cell contact facilitates conjugation (and presumably lethal zygosis) [50, 51]. Furthermore, this mechanism likely functions in concert with other plasmid-encoded arsenals like diffusible toxins (e.g. bacteriocins) [52, 53]. Recent experimental and modeling work has found that the combination of contact-dependent and contact-independent weapons significantly enhances a strain’s competitive ability to defend and invade a niche [27]. Together, these data suggest that lethal zygosis may contribute to plasmid maintenance in the absence of external selection (i.e. the “plasmid paradox” [3]) by providing a selective advantage to plasmid carriers under high-density conditions.

Finally, we applied our findings to synthetic biology. Standard mobilizable vectors inadvertently mimic the conditions for lethal zygosis: they engage the T4SS to deliver DNA but lack exclusion genes, thereby leaving recipients susceptible to conjugation-associated fitness reductions. To resolve this, we sought to improve conjugal delivery by engineering a Mariner transposon to co-deliver the RK2 exclusion gene, *trbK*. Providing this “shield” not only improved transposition efficiency but also protected recipient populations from lethal zygosis (Fig. 3B and Supplementary Fig. S5A). This strategy proved critical for the soil bacterium *Lysobacter*, where standard vectors failed to yield a single edit, but successful editing was achieved when the recipient was protected by *trbK*. These results suggest that some microbes deemed “intractable” may not be recalcitrant to genetic tools, but rather sensitive to lethal zygosis induced by their delivery. The incorporation of exclusion genes into suicide editing vectors could improve our ability to edit previously intractable species. The taxonomic range over which RK2 TrbK can provide this protection remains unknown. However, given the exceptionally broad host range of the RK2/RP4 conjugation system, which can mediate DNA transfer across diverse Gram-negative bacteria and even to Gram-positive and eukaryotic recipients, TrbK may provide protection across a broad range of target organisms [48, 54]. Determining the taxonomic range over which TrbK remains functional will be important for establishing the general applicability of this approach.

Building on these findings, we significantly improved the targeted integration yield of *Vch*CAST by optimizing its recipient-specific expression and co-delivering *trbK* (Fig. 3C). We anticipate that these design principles will be especially critical for *in situ* microbiome editing, where non-replicative vectors are often desirable to avoid the uncontrolled transmission and persistence of replicative plasmids within environmental or host-associated communities [21, 55]. In such complex, real-world settings, donor cells will likely need to be introduced in quantities that far outnumber the targeted recipients to achieve sufficient editing coverage. Our findings demonstrate that these high donor-to-recipient ratios can strongly amplify lethal zygosis, potentially limiting editing yield when exclusion genes are absent. More speculatively, coupling exclusion-gene expression to successful editing could protect edited cells from lethal zygosis while leaving unedited cells susceptible, potentially providing a means to enrich edited cells without additional selection.

This study has several limitations that warrant further investigation. Our work focused on the broad host range RK2 plasmid with hosts within the Gammaproteobacteria. Plasmids with narrower host ranges, stricter conjugation regulation, or anti-defense systems (e.g. anti-CRISPRs, anti-restriction proteins) may exhibit distinct ecological dynamics, and the extent of lethal zygosis could differ substantially [36, 56]. Additionally, we performed our competition assays under simplified, nutrient-rich, aerobic conditions that do not fully recapitulate the metabolic and ecological stresses cells face in natural environments. Finally, we examined only immune systems that recognize and target specific DNA motifs (CRISPR–Cas and RM). Defense systems that respond to other signals like changes to the cell membrane or intracellular metabolite pools may impose different selective pressures [57, 58]. Recently described anti-plasmid defense systems act through substantially different mechanisms, including plasmid clearance and responses triggered by components of the conjugative T4SS [59, 60]. Some of these systems induce growth arrest or loss of viability following plasmid acquisition, raising the possibility that different modes of plasmid recognition could interact with repeated T4SS engagement in distinct ways. Defense systems are not necessarily adaptations of the bacterial host; many are encoded by MGEs themselves and can mediate competition among mobile elements [29, 61, 62]. More broadly, immune system liabilities are not unique to conjugation; defense systems targeting temperate phage can induce lethal autoimmunity, significantly reducing lysogen fitness, and in some circumstances, select for immune system loss [63]. Future work should probe coercive assimilation across a broader diversity of organisms and within their native microbiomes and environments.

Our experiments quantify lethal zygosis primarily through changes in recoverable recipient abundance and competitive fitness and therefore do not directly resolve the cellular basis of this phenotype. Reduced CFU could reflect cell death, but could also be influenced by growth inhibition, altered plating efficiency, or other physiological responses to repeated T4SS engagement [64, 65]. However, recent studies using orthogonal approaches provide direct evidence that excessive conjugative interactions can compromise recipient viability. Couturier *et al*. used single-cell microscopy and viability staining to show that unregulated F-plasmid transfer in the absence of exclusion causes membrane stress, filamentation, loss of membrane integrity, and cell death [6]. Similarly, previous work with the RK2 T4SS demonstrated T4SS-dependent compromised membrane integrity, supporting a lethal cellular phenotype associated with this system [12]. While our results establish a strong T4SS-dependent fitness collapse that is prevented by entry exclusion, direct single-cell measurements would be needed to determine the relative contributions of cell death and other physiological outcomes under our experimental conditions.

Plasmids have long been recognized as key vectors of virulence and antibiotic resistance, but our understanding of their ecological impact has been mostly limited to the accessory genes they carry and the physiological costs they impose on hosts [65–67]. Our findings expand this view by revealing that conjugative plasmids can act as direct ecological agents, restructuring microbial populations through contact-dependent antagonism. More broadly, understanding how plasmids drive this coercive assimilation to shape the tradeoffs between immunity, permissiveness, and horizontal gene transfer will be essential for predicting microbiome dynamics and developing more effective genetic tools.

## Supplementary Material

gkag1043_Supplemental_Files

## Data Availability

Plasmid sequences, maps, code, and raw data are available at https://doi.org/10.5061/dryad.mpg4f4rfm. Materials are available upon reasonable request.
