# Supplementary material for: DNA defense systems become a fitness liability during plasmid conjugation
Source: Nucleic Acids Res. 2026 Oct 29;54(20):gkag1043. doi: 10.1093/nar/gkag1043 (PMC13717191; doi:10.1093/nar/gkag1043)
Supplement: Supplementary file 1 [file gkag1043_supplemental_files.zip › Martinson_NAR_Supplementary_Figures.pdf]

SUPPLEMENTARY DATA

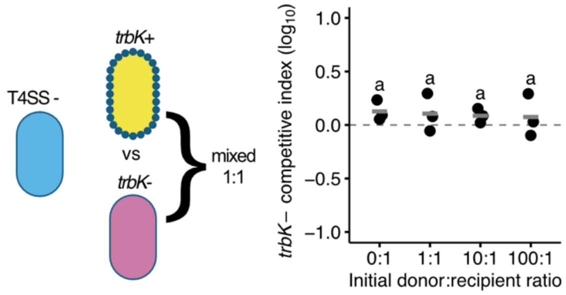

Supplementary Figure 1. Donor-cell crowding alone does not reduce the fitness of recipients lacking entry exclusion. *trbK*– and *trbK*+ recipients were mixed at a 1:1 ratio and competed in the presence of increasing initial ratios of a matched donor-background strain lacking the T4SS (T4SS-). Competitive index represents the relative fitness of *trbK*– recipients compared with *trbK*+ recipients. Points represent biological replicates and gray bars indicate means (n = 3). Different letters indicate significantly different groups (Tukey-adjusted pairwise comparisons, $p < 0.05$); groups sharing a letter are not significantly different. Donor-to-recipient ratio had no detectable effect on *trbK*– recipient fitness (one-way ANOVA, $F(3,8) = 0.076$, $p = 0.971$).

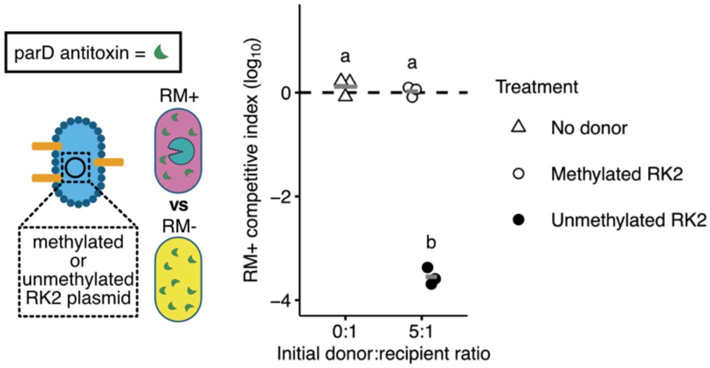

Supplementary Figure 2. Coercive assimilation is not dependent on toxin-antitoxin systems. Recipients with (RM+) or without (RM–) the EcoRII restriction–modification system were pre-expressed with the RK2 parABCDE toxin–antitoxin operon, providing recipients with the cognate ParD antitoxin prior to donor exposure. RM+ and RM– recipients were competed in the absence of donors or in the presence of donors carrying either methylated or unmethylated RK2. Recipient fitness is shown as the $\log_{10}$ competitive index of RM+ relative to RM– cells. Conjugations were performed in biological triplicate, with each point representing an independent biological replicate calculated as the mean of three technical replicates (individual conjugation spots). Treatment significantly affected competitive fitness (one-way ANOVA, $F(2,6) = 612.74$, $P < 0.0001$). Pairwise differences were assessed using Tukey-adjusted multiple comparisons. Letters indicate statistically distinct groups; treatments sharing a letter are not significantly different ($P > 0.05$).

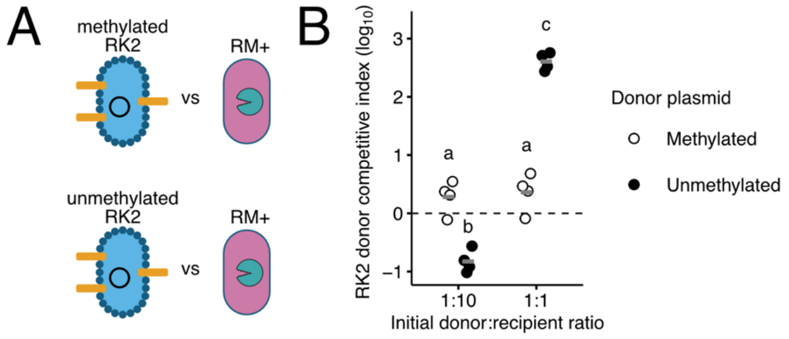

Supplementary Figure 3. RK2 donor fitness depends on initial donor-to-recipient ratio and plasmid methylation. (A) Schematic of the experimental setup. Donors carrying either methylated or unmethylated RK2 were competed against RM+ recipients at different initial donor-to-recipient ratios. (B) Competitive index of RK2 donors at initial donor-to-recipient ratios of 1:10 and 1:1. Open circles indicate donors carrying methylated RK2 and filled circles indicate donors carrying unmethylated RK2. Points represent biological replicates and gray bars indicate means (n = 4). Donor plasmid methylation and initial donor-to-recipient ratio showed a significant interaction (two-way ANOVA, $F(1,12) = 184.17$, $p < 0.0001$). Different letters indicate significantly different groups (Tukey-adjusted pairwise comparisons, $p < 0.05$); groups sharing a letter are not significantly different.

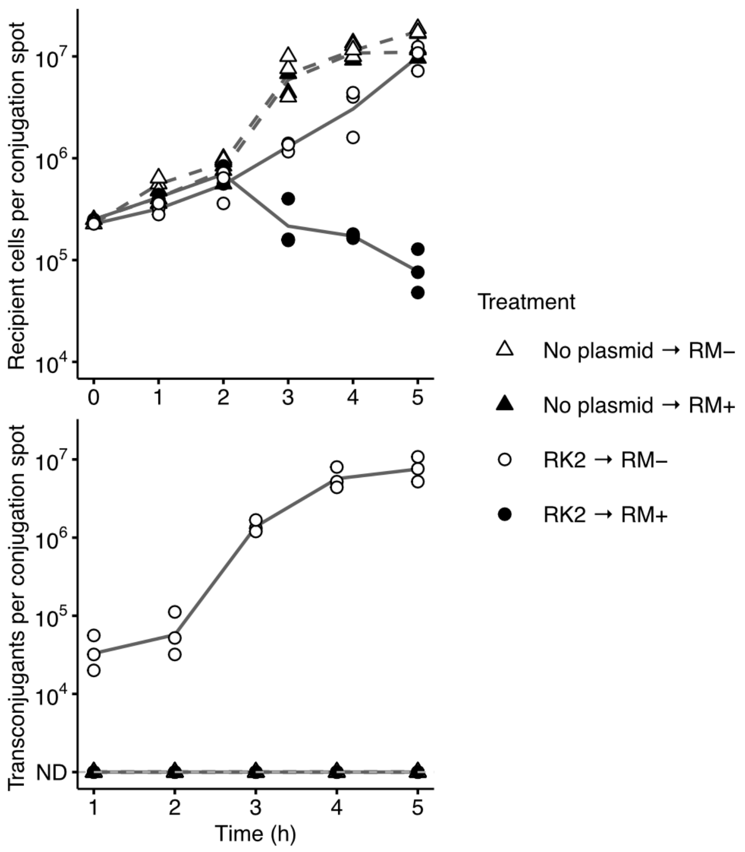

Supplementary Figure 4. Timecourse of conjugation and lethal zygosis. Donor and recipient cells were mixed at a 10:1 donor-to-recipient ratio and conjugated on solid agar for the indicated times. Donor strains either carried the unmethylated conjugative plasmid RK2 (circles) or were plasmid-free strain-background controls (triangles). Recipients either carried the EcoRII restriction–modification (RM) system (RM+, filled symbols) or lacked an RM system (RM–, open symbols). Top: Viable recipient cells recovered per mating spot over time. Bottom: Transconjugants recovered per mating spot; ND, not detected. The experiment represents a single biological replicate, with each point representing an independently prepared conjugation spot destructively sampled at the indicated time. Lines connect the mean of the replicate mating spots at each time point.

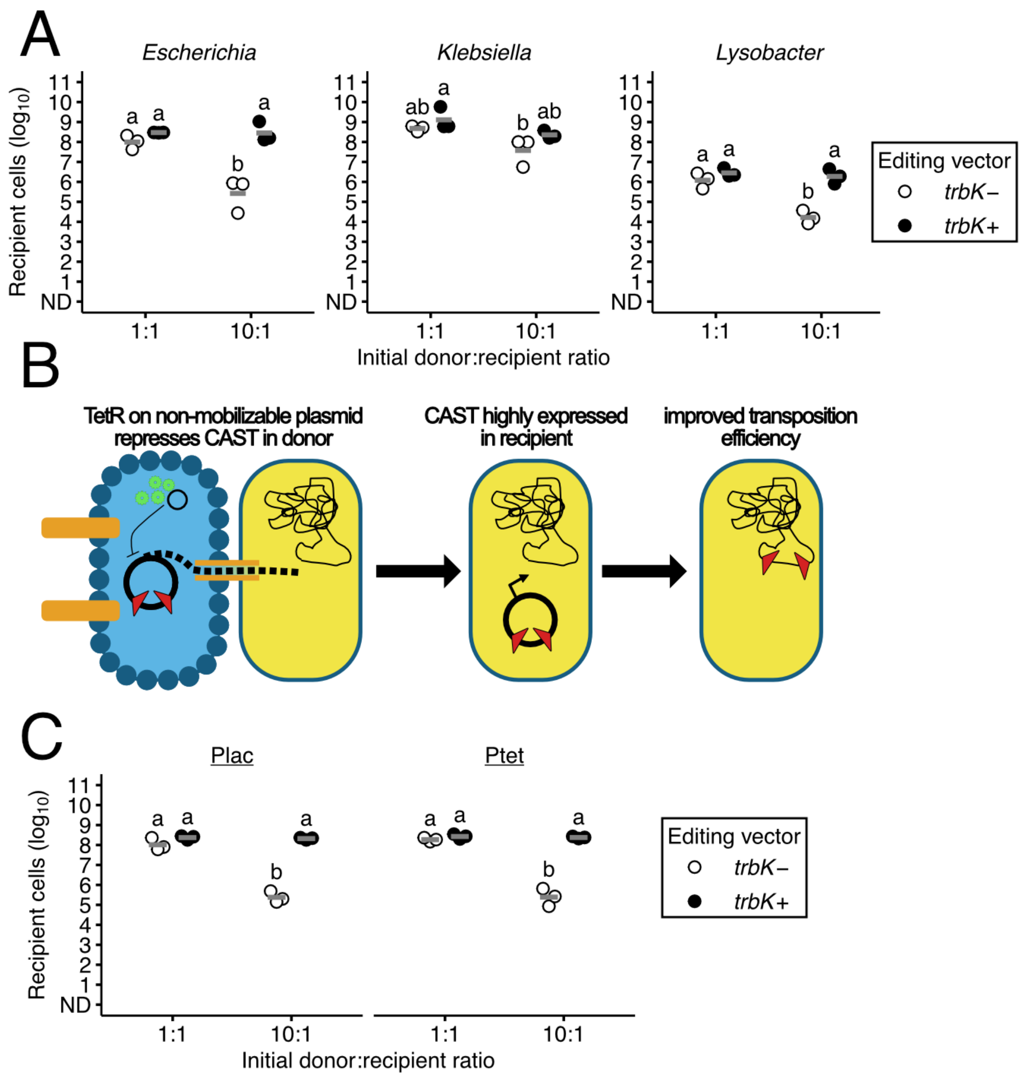

Supplementary Figure 5. Inclusion of exclusion genes shields recipients from lethal zygosis. Total viable recipients following a 5 hour conjugation with **(A)** Mariner transposon vectors delivered to *Escherichia*, *Klebsiella*, and *Lysobacter* recipients at the indicated initial donor-to-recipient ratios. **(B)** Schematic of the split TetR / Ptet *Vch*CAST expression system. (Left) The donor carries a non-mobilizable plasmid expressing the transcriptional repressor, TetR (green cogs), which represses the *Vch*CAST operon under the control of Ptet. (Middle) Upon transfer of the non-replicative vector into the recipient, the absence of TetR permits strong *Vch*CAST expression. (Right) Recipient-specific *Vch*CAST expression improves transposition efficiency. **(C)** Total viable *Escherichia* recipients following conjugation with *Vch*CAST transposon vectors under the control of Plac or Ptet at the indicated donor-to-recipient ratios. For (A) and (C), filled circles indicate editing vectors carrying *trbK* and open circles indicate vectors lacking *trbK*. All conjugations were performed for 5 hours. "ND" indicates that no colonies were detected; values of zero were assigned a pseudocount of 1 prior to $\log_{10}$ transformation for visualization. For **(A)**, data were analyzed separately for each recipient by two-way ANOVA testing the effects of *trbK*, donor-to-recipient ratio, and their interaction. For (C), data were analyzed by three-way ANOVA testing the effects of *trbK*, *Vch*CAST promoter, donor-to-recipient ratio, and their interactions. Different letters indicate significant Tukey-adjusted pairwise differences among conditions ($p < 0.05$); for (C), comparisons include all Plac and Ptet conditions shown. Horizontal bars indicate the mean of biological replicates ($n = 3$).
